# Myocardial Diseases: Current Views on Etiopathogenesis, Diagnostic Modalities, and Therapeutic Options

**DOI:** 10.1155/2016/1720405

**Published:** 2016-06-16

**Authors:** Tomas Palecek, Javier Ganame, Giovanni Di Salvo

**Affiliations:** ^1^2nd Department of Medicine-Department of Cardiovascular Medicine, First Faculty of Medicine, Charles University in Prague and General University Hospital in Prague, Prague, Czech Republic; ^2^McMaster University, Hamilton, ON, Canada; ^3^Heart Center, King Faisal Specialist Hospital and Research Center, Riyadh, Saudi Arabia

According to current European classification, cardiomyopathies are defined as myocardial disorders that cannot be explained by coronary artery disease or abnormal loading conditions including valvular and congenital diseases. Six specific morphological and functional phenotypes are distinguished: hypertrophic, dilated, restrictive, and arrhythmogenic right ventricular cardiomyopathy together with two unclassified subtypes: Tako-tsubo and left ventricular noncompaction cardiomyopathies. In almost all of these phenotypes, inherited forms may be found; moreover, in some of them—for example, in hypertrophic cardiomyopathy—a genetic origin is even a rule. Therefore, genetic counseling shall be an integral part of the state-of-the-art care of patients with heart muscle disorders. The affected individuals together with their relatives have to be informed about the genetic basis of their disease and the potential risk for other family members. A detailed cardiac evaluation, including ECG and echocardiography (in some cases also Holter ECG monitoring), of first-degree relatives is necessary with their further regular follow-up. If available, genetic testing shall be discussed with the patients as it may improve their management as well as enable effective preventive genetic testing in other relatives.

Because the evaluation of morphology and function of the heart, especially of the left ventricle, is a prerequisite for accurate phenotypic characterization of every myocardial disease, a multimodality imaging approach generally becomes necessary. Echocardiography is still a cornerstone of diagnostic imaging because it is accurate, widely available, safe, and without any contraindications. In recent years, advanced echo modalities gained a clinical role in the follow-up of chemotherapy-induced cardiomyopathy, and global longitudinal strain is recommended in the latest European guidelines.

However, the role of cardiac magnetic resonance is increasingly important. Not only is it the current gold standard for morphological and functional assessment of heart chambers, but it has a unique opportunity of noninvasive tissue characterization. Based on evaluation of the presence of myocardial edema, hyperemia, and necrosis using dedicated sequences, cardiac magnetic resonance is capable of diagnosing acute myocarditis with high accuracy. Late gadolinium enhancement technique is widely used to assess the presence and extent of replacement myocardial fibrosis, which appears to be strongly related to the prognosis of affected individuals. Presence of myocardial fibrosis predisposes to increased risk of ventricular arrhythmias, sudden cardiac death, and progression of left ventricular dysfunction associated with heart failure worsening. One also has to mention the significant contribution of various techniques of nuclear medicine for the diagnosis of cardiac sarcoidosis and transthyretin amyloid cardiomyopathy.

It is obvious that increasing knowledge on ethiopathogenesis of various myocardial disorders together with improved diagnostic possibilities shall result in novel therapeutic options. A complex immunohistochemical and PCR analysis of endomyocardial biopsy specimens allows us to characterize in more detail the presence and type of myocardial inflammation as well as the presence of possible causative infectious agents and select better more targeted treatment of acute myocarditis and inflammatory cardiomyopathy with administration of immunosuppressive or immunomodulatory drugs or antiviral agents. Many therapeutic strategies are currently investigated in the field of transthyretin amyloidosis including stabilizers of transthyretin tetramers and gene therapies aimed at suppression of transthyretin expression. Another important topic is the development of new treatment possibilities that will be able to prevent late sequelae of cytostatic therapy associated with potential cardiotoxic effect.

In this special issue we are pleased to introduce several interesting original as well as review articles focused on different topics in the field of myocardial diseases.

- Two original articles deal with the topic of chemotherapy-induced cardiotoxicity. P. Robinson et al. investigated in their experimental study the role of substance P in chemotherapy-associated death of cardiomyocytes and chemoresistance and nicely showed that aprepitant, a substance P receptor antagonist, is able to decrease doxorubicin induced death of cardiomyocytes as well as to increase the sensitivity of cancer cells to this drug. A. F. Yu et al. evaluated the use of 2D speckle tracking echocardiography in early diagnosis of cardiotoxicity associated with anthracyclines and radiation therapy. Their results confirm that this novel imaging modality is more sensitive in detecting left ventricular systolic dysfunction than traditional indices of ventricular function such as ejection fraction and thus can be used for long-term cardiac surveillance of adult cancer survivors.
- Three articles review several aspects of inflammation/infection related myocardial diseases. J. Krejci et al. largely summarize our current knowledge on pathophysiology, diagnosis, and treatment of inflammatory cardiomyopathy with emphasis on accurate evaluation of endomyocardial biopsy samples, which is prerequisite for the appropriate choice of subsequent treatment options. P. Kuchynka et al. nicely overview a difficult topic of eosinophilic myocarditis, a rare disorder necessitating quite rapid diagnosis, so early administration of immunosuppressive therapy that may improve the poor prognosis of affected individuals can be initiated. In the last article, R. H. Lumsden and G. S. Bloomfield present a brilliant review on HIV-associated cardiomyopathy which currently represents a significant cause of morbidity and mortality in this now chronic disease. The authors clearly demonstrate that there is a significant need to design clear guidelines for screening protocols and diagnostic criteria for HIV-related myocardial disease.
- Several issues related to hypertrophic cardiomyopathy are discussed in three articles. R. Pudil et al. aimed to investigate the significance of vascular endothelial growth factor in hypertrophic cardiomyopathy and their results show that increased levels of this substance are associated with structural and functional parameters in patients with hypertrophic cardiomyopathy suggesting the possibility of its use for a more accurate diagnosis. L. K. Williams et al. focused on left atrial function in hypertrophic cardiomyopathy. Using magnetic resonance velocity vector imaging, they convincingly demonstrate a negative impact of left ventricular outflow tract obstruction on left atrial size and function that significantly improves after septal myectomy. Finally, P. P. Dimitrow and R. Rajtar-Salwa review several aspects related to left ventricular outflow tract obstruction with emphasis on invasive as well as noninvasive treatment possibilities.

If the readers will find the presented articles informative and stimulating their interest in myocardial disorders, the purpose of this special issue will be completely fulfilled.

*Tomas Palecek* *Javier Ganame* *Giovanni Di Salvo*

